# Deletion of the Natural Killer Cell Receptor NKG2C Encoding *KLR2C* Gene and Kidney Transplant Outcome

**DOI:** 10.3389/fimmu.2022.829228

**Published:** 2022-03-24

**Authors:** Hannes Vietzen, Bernd Döhler, Thuong Hien Tran, Caner Süsal, Philip F. Halloran, Farsad Eskandary, Carsten T. Herz, Katharina A. Mayer, Nicolas Kozakowski, Markus Wahrmann, Sarah Ely, Susanne Haindl, Elisabeth Puchhammer-Stöckl, Georg A. Böhmig

**Affiliations:** ^1^ Center for Virology, Medical University of Vienna, Vienna, Austria; ^2^ Institute of Immunology, Heidelberg University Hospital, Heidelberg, Germany; ^3^ Transplant Immunology Research Center of Excellence, Koç Üniversitesi, Istanbul, Turkey; ^4^ Alberta Transplant Applied Genomics Centre (ATAGC), University of Alberta, Edmonton, AB, Canada; ^5^ Division of Nephrology and Dialysis, Department of Medicine III, Medical University of Vienna, Vienna, Austria; ^6^ Department of Pathology, Medical University of Vienna, Vienna, Austria; ^7^ Department of Clinical Pharmacology, Medical University of Vienna, Vienna, Austria

**Keywords:** antibody-mediated rejection, donor-specific antibody, kidney transplantation, microvascular inflammation, natural killer cell, NKG2C receptor

## Abstract

Natural killer (NK) cells may contribute to antibody-mediated rejection (ABMR) of renal allografts. The role of distinct NK cell subsets in this specific context, such as NK cells expressing the activating receptor NKG2C, is unknown. Our aim was to investigate whether *KLRC2* gene deletion variants which determine NKG2C expression affect the pathogenicity of donor-specific antibodies (DSA) and, if so, influence long-term graft survival. We genotyped the *KLRC2* ^wt/del^ variants for two distinct kidney transplant cohorts, (i) a cross-sectional cohort of 86 recipients who, on the basis of a positive post-transplant DSA result, all underwent allograft biopsies, and (ii) 1,860 recipients of a deceased donor renal allograft randomly selected from the Collaborative Transplant Study (CTS) database. In the DSA+ patient cohort, *KLRC2* ^wt/wt^ (80%) was associated with antibody-mediated rejection (ABMR; 65% versus 29% among *KLRC2* ^wt/del^ subjects; *P*=0.012), microvascular inflammation [MVI; median g+ptc score: 2 (interquartile range: 0-4) versus 0 (0-1), *P*=0.002], a molecular classifier of ABMR [0.41 (0.14-0.72) versus 0.10 (0.07-0.27), *P*=0.001], and elevated NK cell-related transcripts (*P*=0.017). In combined analyses of *KLRC2* variants and a functional polymorphism in the Fc gamma receptor IIIA gene (*FCGR3A*-V/F158), ABMR rates and activity gradually increased with the number of risk genotypes. In DSA+ and CTS cohorts, however, the *KLRC2* ^wt/wt^ variant did not impact long-term death-censored graft survival, also when combined with the *FCGR3A-*V158 risk variant. *KLRC2* ^wt/wt^ may be associated with DSA-triggered MVI and ABMR-associated gene expression patterns, but the findings observed in a highly selected cohort of DSA+ patients did not translate into meaningful graft survival differences in a large multicenter kidney transplant cohort not selected for HLA sensitization.

## Introduction

Natural killer (NK) cells are well known to play a decisive role as a first line of defence against pathogens and tumour cells, contributing both to innate and adaptive immunity (1). More recent studies have also suggested that NK cells make an important contribution to the rejection of organ allografts (2, 3). The extent of intra-graft NK cell infiltrates was shown to strongly predict graft survival and even to outperform conventional histologic rejection criteria (4). Moreover, in the specific context of anti-HLA donor-specific antibody (DSA)-triggered antibody-mediated rejection (ABMR), gene expression studies have suggested that NK cells activated *via* Fc gamma receptor (FcγR) IIIA may play a pathogenic role (5–8). In line with these results, two recent studies have suggested a relationship between a functional single nucleotide polymorphism (SNP) in the FcγRIIIA gene (*FCGR3A*-V/F158) with microvascular inflammation (MVI), a prominent morphologic lesion in ABMR (9, 10). Nevertheless, in a subsequent analysis of approximately 2,000 kidney transplant recipients, we failed to demonstrate any impact of this SNP on long-term death-censored graft survival, presumably because of the overwhelming detrimental impact of other relevant immunological and non-immunological injury mechanisms (11). Beyond antibody-dependent cellular cytotoxicity (ADCC), alternative DSA-independent pathways of NK cell activation may contribute to transplant injury. These include missing-self mechanisms, where mismatches between recipient inhibitory killer-cell immunoglobulin-like receptors (KIRs) and graft HLA class I molecules may lead to NK cell-driven MVI (12, 13).

NK cells are well known to be regulated through the integrated balance of signalling *via* a panel of different inhibitory and activating receptors, some of them interacting with HLA class I molecules as critical immune checkpoints (1). One activating receptor that could potentially contribute to NK cell-driven alloimmune injury, is the C-type lectin NKG2C (CD159c), a type II integral membrane protein encoded by the *KLRC2* gene located in the NK complex on chromosome 12p13. NKG2C covalently assembles with CD94. The CD94/NKG2C heterodimers bind specifically to HLA-E molecules, which are stabilized by human or viral peptides on the surface of stressed or infected cells. Receptor binding triggers cytotoxic responses and the release of pro-inflammatory molecules, thus promoting the ‘adaptive’ differentiation and expansion of NKG2C+ NK cells (14). The interaction of CD94/NKG2C with HLA-E requires the binding of viral (e.g. CMV-derived) peptides or leader sequences of classical and non-classical HLA molecules. It was shown that the leader peptide of the non-classical HLA-G molecule VMAPRTLFL is a strong HLA-mediated activator of CD94/NKG2C mediated cytotoxicity and proliferation of NKG2C+ NK cells (15). In a recently published study, using a Puumala virus model, we could demonstrate, that the cellular stress responses and upregulation of HLA-G is indeed sufficient to induce a potent HLA-E-mediated cytotoxic NKG2C+ NK cell response (16).

The level of NKG2C expression may be determined by a distinct polymorphism leading to homo- (absent expression) or heterozygous (lower expression) deletion of the *KLRC2* gene, which in different populations was found in up to 2% and 33% of tested individuals, respectively (17). This genetic variation has a strong impact on the number and functionality of NKG2C+ NK cells (18–20). Clinical association studies have suggested that NKG2C copy number is related to the susceptibility and/or severity of different viral infections, as has been shown for cytomegalovirus (CMV) in lung transplant recipients (21), HIV (22) and SARS-CoV-2 (17).

There is some evidence that NKG2C+ NK cells contribute to transplant rejection, as has recently been shown for recipients of lung allografts (23). The role of NKG2C expression or *KLRC2* gene variations in kidney transplantation, however, has not yet been investigated. Hypothesizing a role of NKG2C+ NK cells in the context of antibody-dependent and -independent alloresponses, we sought to investigate whether and to which extent deletion of the *KLRC2* gene protects allografts from DSA-triggered microcirculation injury. Moreover, we were interested, if there is any additive effect of a functional SNP in the FcγRIIIA gene (*FCGR3A*-V/F158). In a first step, we studied a small well-characterized cohort of 86 DSA+ renal allograft recipients, who, late after transplantation, underwent biopsies for detailed histomorphologic and molecular assessment. This allowed us to thoroughly study the role of NKG2C expression in the specific context of DSA-triggered inflammation and injury. In a second step, we analyzed long-term graft outcomes in relation to *KLRC2* variants in a large, randomly selected multicenter prospective cohort of 1,860 recipients of deceased donor kidney transplants.

## Materials and Methods

### Study Design and Patient Cohorts

**Figure 1:**
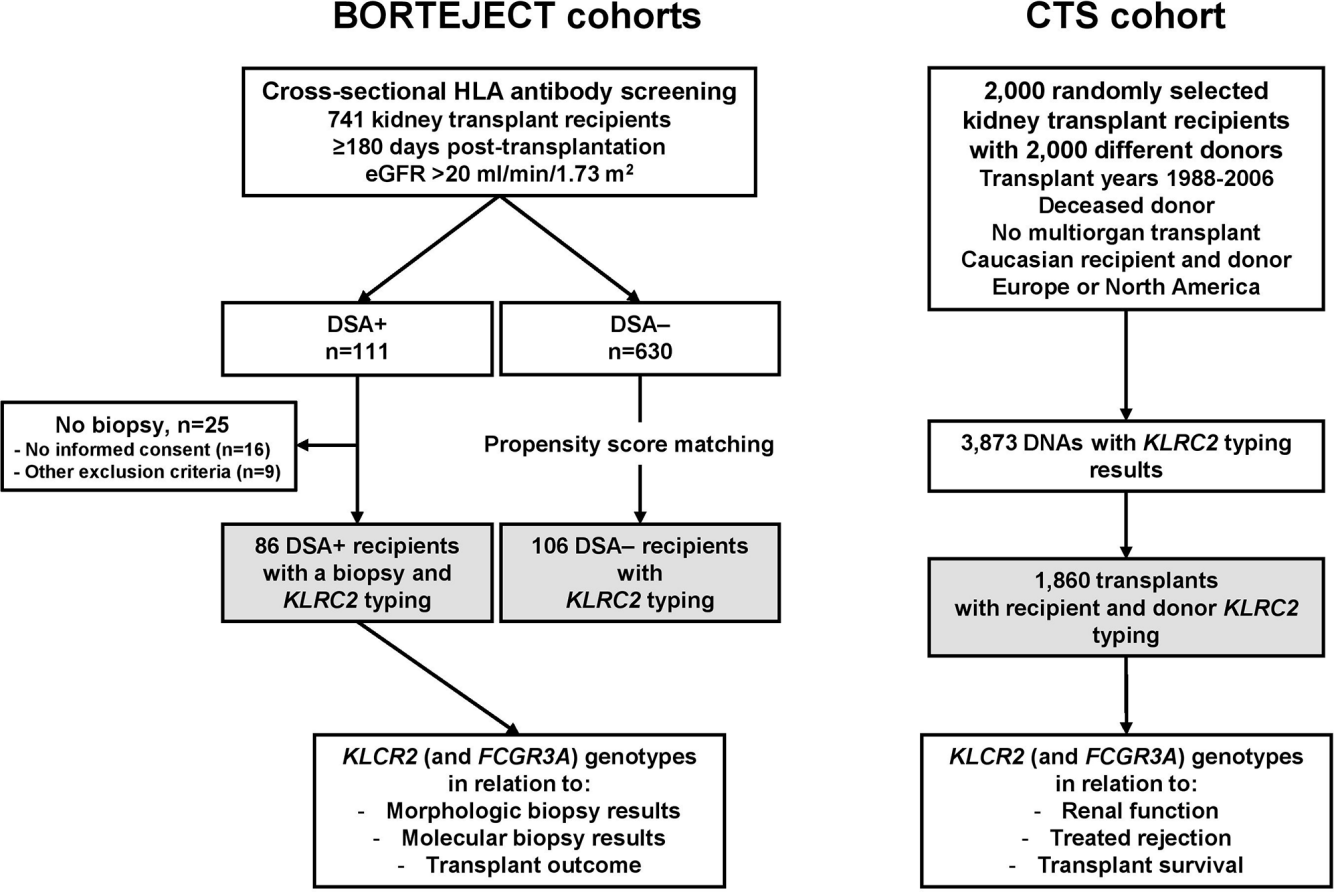
Study flow chart. Systematic cross-sectional antibody-mediated rejection (ABMR) screening of a cohort of 741 kidney transplant recipients (BORTEJECT trial) led to the identification of 111 donor-specific antibody (DSA)-positive recipients. Eighty-six DSA+ patients underwent protocol biopsies. For all 86 subjects (primary study cohort) adequate material for *KLRC2* genotyping was available. A control group of 106 recipients was defined by propensity score matching as described in the methods section. A large prospective multicenter cohort (Collaborative Transplant Study, CTS; 1,860 recipient/donor pairs) was included to assess associations of genotyping results in relation to long-term allograft outcomes. eGFR, estimated glomerular filtration rate.

The study included two independent prospective transplant cohorts, (i) a cohort of 86 kidney transplant recipients [BORTEJECT cohort (24)] who, based on a positive post-transplant DSA result, underwent allograft biopsies, and (ii) a large cohort of randomly selected recipient/donor pairs from the multicenter Collaborative Transplant Study (CTS, www.ctstransplant.org). A flow chart of the study is provided in **Figure 1**. Genotyping and statistical data analysis were carried out in a retrospective and blinded fashion.

The first cohort (86 DSA+ recipients) was recruited *via* post-transplant DSA screening for a randomized controlled interventional trial to evaluate the effect of the proteasome inhibitor bortezomib in late ABMR (BORTEJECT, ClinicalTrials.gov: NCT01873157) (24). Briefly, 741 recipients in outpatient care (key inclusion criteria: age >18 years, ≥180 days post-transplantation, estimated glomerular filtration rate [eGFR] >20 ml/min per 1.73 m^2^) underwent cross-sectional HLA antibody screening (October 2013 through February 2015). The study cohort consisted of 86 patients who were DSA+ and underwent systematic allograft biopsies. Baseline characteristics are provided in **Table 1**. For all recipients, sufficient material was available for *KLRC2* genotyping (*FCGR3A* genotyping: n=85). For analysis of *KLRC2* genotype distributions in relation to DSA status, we selected 106 of the 630 DSA– recipients. For DSA– recipients, no protocol biopsies were available. As detailed in a previous study (25), these subjects were propensity score matched with the DSA+ study patients on the basis of female sex, recipient age at transplantation, protein/creatinine ratio (PCR), prior transplantation, HLA mismatch, and cytotoxic panel reactivity. As shown in **Supplementary Table 1**, DSA+ and DSA– cohorts did not significantly differ with respect to baseline data, with the exception of more frequent recipient desensitization among DSA+ patients.

**Table 1:** Baseline characteristics - cohort 1 (BORTEJECT trial).

Parameters	All recipients	*KLRC2* ^wt/wt^	*KLRC2* ^wt/del^	*P* value
	n=86	n=69	n=17	
**Characteristics obtained at the time of transplantation**				
Female recipient sex, n (%)	39 (45)	33 (48)	6 (36)	0.42
Recipient age (years), median (IQR)	47.3 (35.8–54.0)	47.7 (35.1–54.1)	46.2 (40.7–54.8)	0.63
First renal allograft, n (%)	61 (71)	48 (70)	13 (76)	0.77
Living donor transplantation, n (%)	14 (16)	12 (17)	2 (12)	0.74
Donor age (years)**^a^**, median (IQR)	46 (35–58)	46 (36–58)	43 (27–56)	0.49
Cold ischemia time (hours)**^a^**, median (IQR)	12.0 (8.5–17.2)	12.0 (8.7–17.3)	12.3 (4.2–16.0)	0.72
HLA mismatch (A, B, DR)**^a^**, median (IQR)	3 (2-4)	3 (2-4)	3 (2-4)	0.51
CDC-PRA >0%**^a^,** n (%)	30 (37)	25 (38)	5 (33)	>0.99
Preformed DSA**^b^,** n (%)	25 (60)	24 (71)	1 (13)	0.004
ABO-incompatible living donor transplant**^c^**, n (%)	1 (1)	1 (1)	0 (0)	>0.99
Recipient desensitization, n (%)	26 (30)	23 (33)	3 (18)	0.25
CDC crossmatch conversion, n (%)	8 (9)	7 (10)	1 (6)	>0.99
CMV status**^a^**, n (%)				0.60
R–/D+	11 (13)	10 (15)	1 (6)	
R+/D+ or R+/D–	58 (70)	45 (68)	13 (77)	
R–/D–	14 (17)	11 (17)	3 (18)	
**Characteristics obtained at the time of ABMR screening**				
Time to screening (years), median (IQR)	4.9 (1.9–12.8)	5.0 (2.0–13.2)	4.6 (0.9–12.7)	0.49
Serum creatinine (mg/dL), median (IQR)	1.6 (1.2–2.1)	1.5 (1.2–2.0)	1.6 (1.3–2.1)	0.63
eGFR (mL/min/1.73m^2^), median (IQR)	54 (32–79)	54 (31–84)	52 (32–65)	0.78
Urinary protein/creatinine ratio (mg/g), median (IQR)	192 (79–445)	216 (85–571)	141 (70–215)	0.093
Maintenance immunosuppression				
Triple immunosuppression	65 (76)	52 (75)	13 (77)	>0.99
Dual immunosuppression	21 (24)	17 (25)	4 (24)	>0.99
Tacrolimus	52 (60)	43 (62)	9 (53)	0.58
Cyclosporine A	29 (34)	22 (32)	7 (41)	0.57
mTOR inhibitor	4 (5)	3 (4)	1 (6)	>0.99
Belatacept	1 (1)	1 (1)	0	>0.99
Mycophenolic acid	71 (83)	57 (83)	14 (82)	>0.99
Azathioprine	5 (6)	4 (6)	1 (6)	>0.99
Steroids	75 (88)	60 (87)	15 (88)	>0.99

CDC, complement-dependent cytotoxicity; DSA, donor-specific antibody; eGFR, estimated glomerular filtration rate; HLA, human leukocyte antigen; IQR, interquartile range; mTOR, mammalian target of rapamycin; PRA, panel-reactive antibody.

**^a^** Donor age, cold ischemia time, HLA mismatch, CDC panel reactivity and CMV status were not recorded for 3, 5, 1, 5, and 3 recipients, respectively.

**^b^** Pre-transplant single antigen testing was available for 42 patients (solid-phase HLA antibody screening on the wait list according to our local standard implemented in July 2009). Pre-sensitized patients (until 2009: ≥40% CDC-PRA; since 2009: preformed DSA) were subjected to a protocol of peri-transplant immunoadsorption as earlier detailed.

**^c^** This patient underwent desensitization for ABO (AB donor to O recipient) plus HLA antibody (DSA+) barriers.

The second cohort, which has been previously described in detail (11, 26), was randomly selected from the CTS database using the following inclusion criteria: single-organ deceased donor kidney transplantation between 1988 and 2006 in Europe or Northern America; Caucasian recipient and donor; complete database record of original disease, recipient and donor age, graft number, or HLA-A, -B, -DR typing; and known immunosuppression scheme. Initially, 2,070 recipient-donor pairs were randomly chosen from the DNA bank of the CTS study. For 3,873 of the selected samples, sufficient DNA was available, and complete genotyping was possible for both recipients and donors (no pairing of one donor with two recipients), resulting in a total of 1,860 transplants from 52 transplant centers in 13 countries finally being included in this study. Baseline characteristics are provided in **Table 2**.

**Table 2:** Baseline characteristics - cohort 2 (Collaborative Cohort Study, Heidelberg).

Characteristics	All recipients	*KLRC2* ^wt/wt^	*KLRC2* ^wt/del^	NKG2C^del/del^	*P* value
	n=1,860	n=1,247	n=530	n=83	
Female recipient sex, n (%)	725 (39)	481 (39)	215 (41)	29 (35)	0.54
Recipient age, mean ± SD (years)	46.6 ± 13.8	46.7 ± 13.8	46.2 ± 14.0	47.5 ± 12.7	0.62
Geographic origin, n (%)					0.29
Europe	1,646 (88)	1,107 (89)	470 (89)	69 (83)	
Northern America	214 (12)	140 (11)	60 (11)	14 (7)	
First renal allograft, n (%)	1,620 (87)	1,077 (86)	469 (88)	74 (89)	0.40
Underlying renal disease, n (%)					0.86
Glomerulonephritis	593 (32)	404 (32)	165 (31)	24 (29)	
Polycystic kidneys	236 (13)	153 (12)	72 (14)	11 (13)	
Diabetes mellitus	171 (9)	120 (10)	42 (8)	9 (11)	
Other	860 (46)	570 (46)	251 (47)	39 (47)	
Donor age, mean ± SD (years)	40.3 ± 16.6	40.1 ± 16.7	40.7 ± 16.3	41.3 ± 16.0	0.65
Cold ischemia time, mean ± SD (hours)	20.5 ± 8.3	20.1 ± 8.1	21.2 ± 8.1	21.8 ± 11.5	0.021
HLA A+B+DR mismatches, n (%)					0.087
0 – 1	217 (12)	134 (11)	76 (14)	7 (8)	
2 – 4	1,384 (74)	948 (76)	375 (71)	61 (73)	
5 – 6	259 (14)	165 (13)	79 (15)	15 (18)	
Panel-reactive antibodies >0%^a^, n (%)	412 (24)	283 (24)	115 (23)	14 (19)	0.56
Initial immunosuppression, n (%)					
Calcineurin inhibitor, n (%)					0.82
Cyclosporine A	1,509 (81)	1,008 (81)	430 (81)	71 (86)	
Tacrolimus	261 (14)	177 (14)	76 (14)	8 (10)	
None	90 (5)	62 (5)	24 (5)	4 (5)	
Antimetabolite agent, n (%)					0.016
Azathioprine	903 (47)	579 (46)	286 (54)	43 (52)	
Mycophenolic acid	648 (33)	404 (32)	144 (27)	30 (36)	
None	389 (20)	264 (21)	100 (19)	10 (12)	
Induction therapy, n (%)					0.98
IL-2R antibody	139 (8)	90 (8)	42 (8)	7 (9)	
Depleting anti-lymphocyte agent	412 (23)	277 (24)	117 (23)	18 (24)	
None	1,208 (69)	811 (69)	347 (69)	50 (67)	

SD, standard deviation; HLA, human leukocyte antigen; IL-2R, interleukin 2 receptor.

a For 131 (7%) recipients, levels of panel-reactive antibodies were not available.

For both cohorts, biologic material and clinical data had been collected prospectively, after written informed consent had been obtained (BORTEJECT cohort: ethics committee of the Medical University of Vienna; CTS cohort: local ethics committees of contributing transplantation centers in Europe and Northern America). Genotyping of included CTS samples was approved by the ethics committee of the University of Heidelberg under the application number 083/2005. The study was conducted in accordance with the Good Clinical Practice Guidelines, the principles of the Declaration of Helsinki 2008, and the Declaration of Istanbul.

### NKG2C (*KLRC2*) and FcγRIIIA (*FCGR3A*) Genotyping

Genomic DNA was purified from peripheral blood of recipients (BORTEJECT; CTS) and from lymph node or spleen samples from donors (CTS). *KLRC2* and *FCGR3A* genotyping were performed following previously described protocols (9, 18). Briefly, *KLRC2* genotyping, was performed by touchdown PCR carried out in 96-well optical plates on a SimpliAmp Thermal Cycler (Thermo-Scientific). Amplicons were visualized on 3% agarose gel containing 0.00005% Midori Green Advance (Nippon Genetics) and analyzed on an iBright CL750 imaging system (Thermo-Scientific). Analysis of the exon polymorphism *FCGR3A*-V/F158 (denoted as rs396991) was carried out in 384-well optical plates on a 7900HT fast real-time PCR system (Applied Biosystems, Rotkreuz, Switzerland) using TaqMan^®^ SNP Genotyping Assay and TaqMan^®^ Universal PCR Master Mix (Fisher Scientific Austria). According to a previously published protocol, two TaqMan^®^ MGB probes were used for each genotyping assay. The first probe detecting allele 1 (Valine/*FCGR3A*) was labelled with VIC dye, and the second probe, labeled with FAM, detected allele 2 (Phenylalanine/*FCGR3A*).

### HLA Antibody Detection

For identification and characterization of DSA, we applied single-antigen flow bead testing (LABScreen Single Antigen assays; One Lambda, A Thermo Fisher Scientific Brand, Canoga Park, CA, USA). Patient sera were heat-inactivated (30 min, 56°C) to preclude complement interference. Thresholds were set at mean fluorescence intensity (MFI) levels >1,000. As previously described in detail (24, 27), donor specificity of HLA reactivity was determined according to the results of serologic and/or low- or high-resolution donor/recipient HLA typing (HLA-A, -B, -Cw, -DR, -DQ, and/or -DP). Test results were documented as the MFI of the immunodominant DSA.

### Transplant Biopsies

The work-up of renal allograft biopsies obtained within the BORTEJECT trial has previously been described in detail (24). Biopsies were re-scored and subcategorized following the 2017 update to the Banff scheme (28). ABMR was defined on the basis of histomorphologic, immunohistochemical (C4d), ultrastructural (multilayering of peritubular capillary basement membranes), and serologic (DSA detection) results. In addition, we included a validated molecular classifier for ABMR (molecular ABMR score ≥0.2) using the Molecular Microscope Diagnostic System (MMDx) (29). ABMR-related single lesions, glomerulitis (g), peritubular capillaritis (ptc) and glomerular basement membrane double contours (cg), were scored according to the Banff rules. MVI was quantified using a g+ptc sum score. For 83 of the 86 index biopsies obtained for the BORTEJECT cohort, a thorough analysis of gene expression patterns was available. The following process, which has previously been described in detail (24) was used for gene expression analysis: A 3-mm portion of a biopsy core was placed in RNAlater, stored at -20°C and shipped at room temperature to the Alberta Transplant Applied Genomics Centre (ATAGC, University of Alberta, Edmonton, AB, Canada). RNA extraction and gene expression analysis were performed using PrimeView GeneChip arrays (Affymetrix Santa Clara, CA, USA). A thorough analysis of gene expression patterns included an actualized re-evaluation of classifiers related to rejection (ABMR, ‘all rejection’), archetypal categories of rejection, and pathogenesis-based transcripts (PBT) scores, including NK cell burden (NKB), gamma-interferon (GRIT1), T cell burden (TCB) and macrophage (QMAT, AMAT1) associated transcripts, based on a recently described reference set of 1,679 indication biopsies (30).

### Statistics

Continuous data are presented as median and interquartile range (IQR) or mean and standard deviation (SD), and categorical variables as absolute and relative frequencies. For inter-group comparisons we applied Chi-square test, Fisher’s exact, Mann-Whitney U, or Kruskal-Wallis tests, as appropriate. Kaplan-Meier analysis was used for calculation of graft survival. The Mantel-Cox log-rank test was applied for the comparison of survival between groups. Multivariable Cox regression analyses were adjusted for the following variables: year of transplantation, geographical region (continent), first or re-transplant, recipient and donor sex and age, cold ischemia time, number of HLA A+B+DR MM, preformed panel-reactive antibodies, original disease leading to end stage renal failure, and immunosuppressive therapy (induction, intention to treat: calcineurin inhibitor, antimetabolite, steroid). Hazard ratios (HR) are presented with 95% confidence intervals (CI). A two-sided *P* value less than 0.05 was considered statistically significant. For statistical analysis IBM SPSS Statistics (version 28, SPSS Inc., Chicago, IL, USA) was used.

## Results

To investigate the relationship between NKG2C (*KLRC2*) gene variants and renal allograft outcomes, two distinct independent transplant cohorts were included (**Figure 1**): (i) a single center cohort of 86 kidney transplant recipients who all had a positive post-transplant DSA result and underwent allograft biopsies ≥180 days post-transplantation (cohort 1), and (ii) a multicenter cohort of 1860 donor/recipient pairs randomly selected from the CTS database (cohort 2).

### Cohort 1 – DSA+ Patients Who Underwent Renal Allograft Biopsies

**Figure 2:**
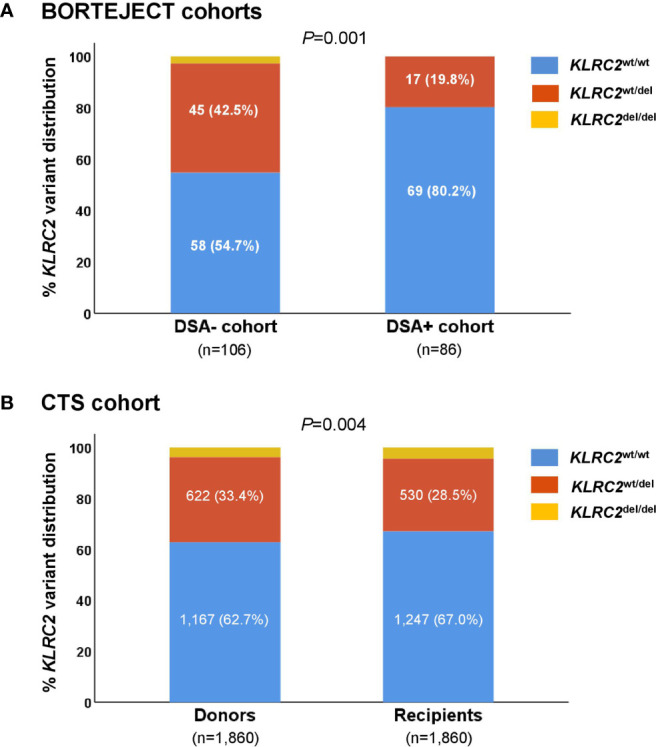
Recipient *KLRC2* variant distribution among **(A)** donor-specific antibody (DSA)+ study patients versus propensity score-matched DSA– control subjects (patients selected from the BORTEJECT trial), and **(B)** donors versus recipients of the Collaborative Transplant Study (CTS) study cohort. Genotype frequencies in the CTS recipient population deviated from Hardy Weinberg equilibrium (*P* = 0.008), but not the donor-specific antibody (DSA)+ (*P* = 0.31), DSA– (*P*=0.10) and CTS donor cohorts (*P* = 0.29), respectively.

As shown in **Table 1**, 39 patients (45%) were female, 61 (71%) had received their first allograft, and 26 (30%) had undergone peri-transplant immunoadsorption for desensitization. Of the 42 recipients subjected to bead array testing before transplantation, 25 (60%) had pre-formed DSA. The majority of the recipients (83%) had high (donor-positive, recipient-negative) or intermediate-risk (recipient-positive) CMV serostatus constellations. Patients underwent ABMR screening a median of 4.9 years post-transplantation. At the time of screening, the majority of recipients were on tacrolimus (60%) and triple immunosuppression (76%) (**Table 1**). As illustrated by **Figure 2**, 69 recipients (80%) encoded for the *KLRC2* ^wt/wt^, and 17 (20%) for the *KLRC2* ^wt/del^ variant. None of the patients were homozygous for *KLRC2* ^del^. The distribution of *KLRC2* variants differed significantly (*P*=0.001) from that found in a matched cohort of 106 DSA– recipients, where the proportion of *KLRC2* ^wt/wt^ patients was lower (55%). Genotype frequencies did not deviate from Hardy-Weinberg equilibrium (**Figure 2**). As detailed in **Table 1**, baseline parameters obtained at the time of transplantation and index biopsy were not significantly different between genotypic variants.

#### KLRC2 Genotype in Relation to Biopsy Results and Transplant Survival

**Figure 3:**
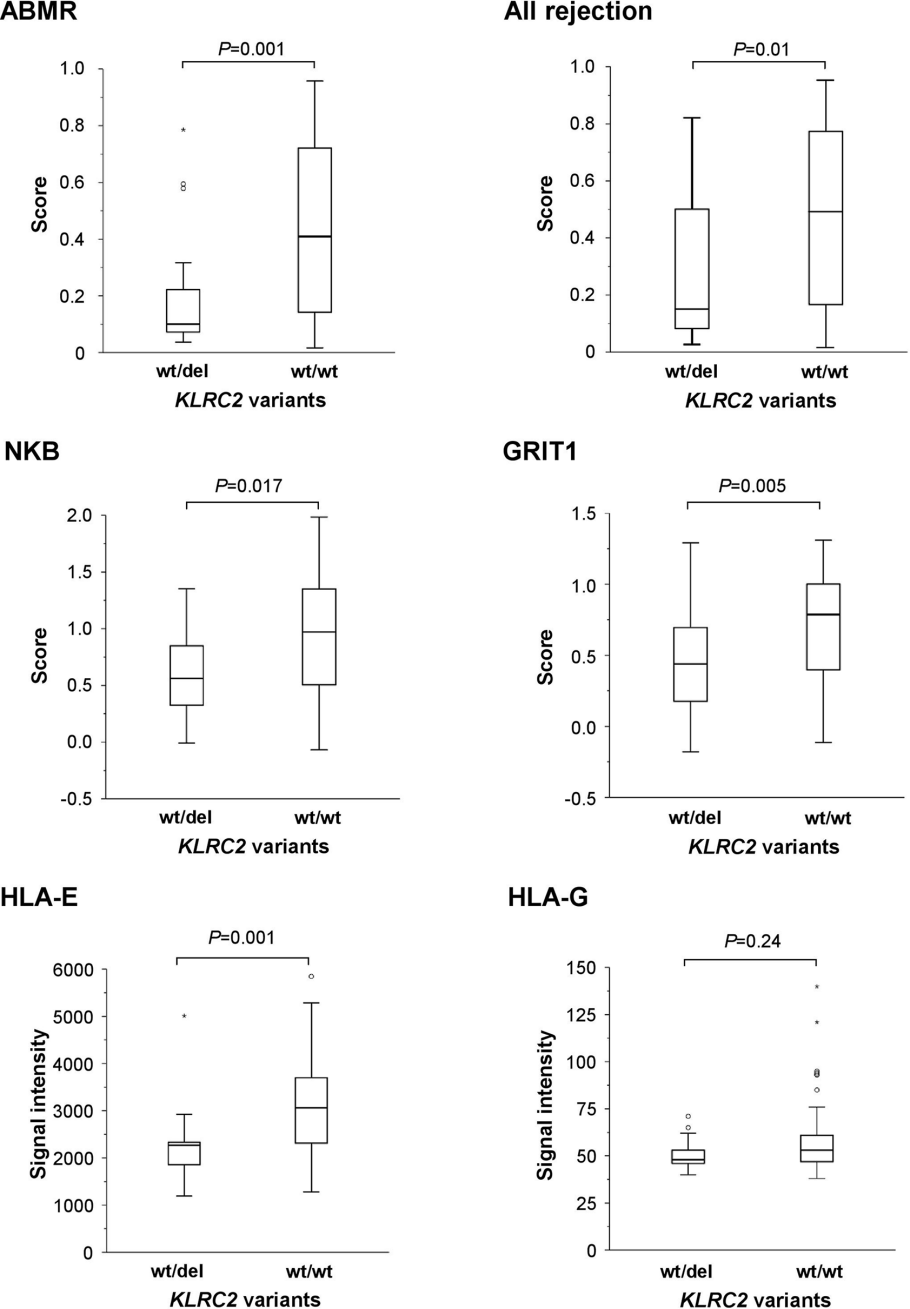
*KLRC2* polymorphism in the DSA+ BORTEJECT cohort in relation to molecular rejection-related scores (ABMR, ‘all rejection’), NK cell burden (NKB)- and IFN-y-related transcripts (GRIT1), or expression of HLA-E and HLA-G (microarray signal intensity). Box plots represent the median, interquartile range and range. Outliers are indicated by circles and extreme outliers by asterisks.

**Figure 4:**
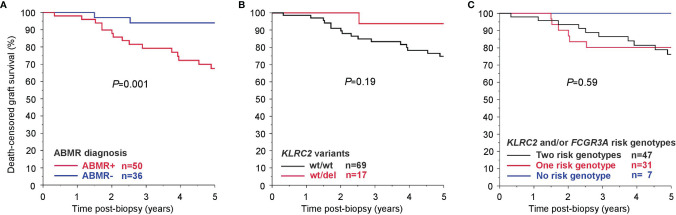
Kaplan Meier death-censored graft survival in the DSA+ cohort (BORTEJECT) in relation to **(A)** antibody-mediated rejection (ABMR) diagnosis, **(B)** *KLRC2* genotype (*KLRC2* ^wt/wt^ vs *KLRC2* ^wt/del^), and **(C)** the number of NKG2C and Fc gamma receptor (FcγR)IIIA ‘risk genotypes’ (*KLRC2* ^wt/wt^; *FCGR3A*-V/V158 or V/F158), respectively.

As shown in **Table 3**, the homozygous *KLRC2* ^wt/wt^ variant was associated with a higher proportion of ABMR as defined by the Banff 2017 scheme (65% versus 29%, *P*=0.012) or molecular archetypal analysis (56% versus 24%, *P*=0.028). In addition, *KLRC2* ^wt/wt^ variant was associated with higher ABMR-related single lesion scores (g, ptc), higher levels of MVI (g+ptc sum score), elevated molecular rejection scores (ABMR score; ‘all rejection’ score), NK cell burden (NKB)- and IFN-y-related transcripts (GRIT1), and increased expression of HLA-E (but not HLA-G) (**Table 3** and **Figure 3**). As illustrated in **Supplementary Figure 1**, also gene expression of classical HLA molecules HLA-A, HLA-B and HLA-DR was significantly increased. At the same time, *KLRC2* ^wt/wt^ recipients had higher DSA MFI, but HLA class specificity and DSA number were not different. As shown in **Table 3**, there was also a marginal increase in macrophage-associated transcripts. In contrast, there were no inter-group differences with respect to morphologic scores related to TCMR and Banff borderline rejection or TCMR and T cell burden related molecular scores (**Table 3**). As shown in **Figure 4**, the histologic diagnosis of ABMR was associated with inferior 5-year kidney allograft survival, but associations between *KLRC2* genotype and rejection-related biopsy findings did not translate into statistically significant graft survival differences (**Figure 4**).

**Table 3:** Rejection-related parameters – cohort 1 (BORTEJECT).

Parameters	DSA+ patients	*KLRC2* ^wt/wt^	*KLRC2* ^wt/del^	*P* - value
	n=86	n=69	n=17	
**DSA characteristics**				
HLA class I DSA, n (%)	27 (31)	21 (30)	6 (35)	0.77
HLA class II DSA, n (%)	42 (49)	35 (51)	7 (41)	0.59
HLA class I+II DSA, n (%)	17 (20)	13 (19)	4 (24)	0.74
MFI of the immunodominant DSA, median (IQR)	2,952 (1,476–7,454)	3,470 (1,755–9,698)	1,474 (1,173–2,188)	0.005
DSA number, median (IQR)	1 (1–2)	1 (1–2)	1 (1–2)	0.46
**Renal allograft biopsy results** ^a^				
Banff 2017 categories				
ABMR, n (%)	50 (58)	45 (65)	5 (29)	0.012
C4d-positive ABMR, n (%)	24 (28)	22 (32)	2 (12)	0.13
Active ABMR, n (%)	15 (17)	14 (20)	1 (6)	0.28
Chronic active ABMR, n (%)	33 (38)	30 (44)	3 (18)	0.057
cg without current/recent Ab interaction, n (%)	2 (2)	1 (1)	1 (6)	0.36
Banff borderline lesion, n (%)	9 (11)	6 (9)	3 (18)	0.37
ABMR-related single lesions				
ptc score^b^, median (IQR)	0 (0–2)	1 (0–2)	0 (0–0)	0.002
g score^b^, median (IQR)	1 (0–2)	1 (0–2)	0 (0–1)	0.026
g+ptc score, median (IQR)	2 (0–3)	2 (0–4)	0 (0–1)	0.002
cg score^b^, median (IQR)	0 (0–1)	0 (0–1)	(0 (0–0)	0.072
TCMR-related single lesions				
t score, median (IQR)	0 (0–0)	0 (0–0)	0 (0–0)	0.45
i score, median (IQR)	0 (0–0)	0 (0–0)	0 (0–1)	0.13
MMDx analysis^c^				
Archetypal analysis				
ABMR archetype, n (%)	41 (49)	37 (56)	4 (24)	0.028
Early onset ABMR, n (%)	13 (16)	10 (15)	3 (18)	0.72
Fully active ABMR, n (%)	24 (29)	23 (35)	1 (6)	0.018
Late ABMR, n (%)	4 (4.8)	4 (6.1)	0 (0)	0.58
TCMR archetype, n (%)	1 (1)	0 (0)	1 (6)	0.21
No rejection archetype, n (%)	41 (49)	29 (44)	12 (71)	0.061
Rejection classifier				
ABMRpm score^c^, median (IQR)	0.36 (0.10–0.65)	0.41 (0.14–0.72)	0.10 (0.07–0.27)	0.001
TCMR score^c^, median (IQR)	0.03 (0.02–0.04)	0.03 (0.02–0.04)	0.03 (0.02–0.04)	0.91
All rejection score^c^, median (IQR)	0.46 (0.13–0.75)	0.49 (0.17–0.78)	0.15 (0.07–0.52)	0.01
Pathogenesis-based transcript (PBT) scores				
NK cell burden-associated (NKB), median (IQR)	0.74 (0.43–1.31)	0.97 (0.50–1.35)	0.56 (0.32–0.87)	0.017
IFN-γ-associated (GRIT1), median (IQR)	0.70 (0.31–0.97)	0.79 (0.39–1.0)	0.44 (0.08–0.70)	0.005
T cell burden (TCB), median (IQR)	1.29 (0.91–1.91)	1.29 (0.98–1.94)	1.42 (0.63–1.67)	0.42
Macrophage-associated transcripts				
QCMAT, median (IQR)	0.35 (0.19–0.57)	0.41 (0.22–0.61)	0.22 (0.10–0.40)	0.032
AMAT1, median (IQR)	0.41 (0.19–0.63	0.45 (0.21–0.65)	0.28 (-0.06–0.49)	0.099

ABMR, antibody-mediated rejection; cg, glomerular basement membrane double contours; DSA, donor-specific antibody; g, glomerulitis; IFN-γ, interferon-gamma; IQR, interquartile range; MFI, mean fluorescence intensity; MMDX, Molecular Microscope Diagnostic System; NK cell, natural killer cell; ptc, peritubular capillaritis; TCMR, T cell-mediated rejection.

a Morphologic lesions were scored according to the Banff 2017 classification of renal pathology.

**^b^** For 2, 4 and 5 recipients, biopsy material was not sufficient for ptc, g and cg scoring, respectively.

c Gene expression analysis was performed in 83 of the 86 study patients.

#### Combined Analysis of KLRC2 and FCGR3A Variants

For 85 of the 86 recipients, results of *FCGR3A*-V/F158 genotyping were available. Eighteen recipients (21%) had V/V158, 40 (47%) V/F158 and 27 (32%) F/F158 genotypes (data not shown). As has been reported previously (9), the 58 patients with one or two high activity F158 alleles presented with higher levels of ptc [1 (0-1) versus 0 (0-1), *P*=0.019] and a higher g+ptc sum score [2 (0-4) versus 0 (0-1), *P*=0.038] than V/V158 recipients (data not shown). In a combined analysis of *KLRC2* and *FCGR3A* variants (**Table 4**), we found a stepwise increase in rates of ABMR diagnosis, microcirculation inflammation, molecular ABMR activity or NKB and GRIT1 PBT with the number of ‘risk genotypes’ (*KLRC2* ^wt/wt^ and *FCGR3A*-V/V158 or V/F158). As shown in **Figure 4**, death-censored graft survival was not different between ‘risk genotype’ groups.

**Table 4:** Combined analysis of NKG2C (*KLRC2)* and FcγRIIIA (*FCGR3A*) genotypes – cohort 1 (BORTEJECT).

	Number of risk genotypes			
	(*KLRC2* ^wt/wt^ and/or *FCGR3A*-V/V158 or -V/F158)			
Parameters	Two risk genotypes	One risk genotype	No risk genotype	*P* value
	n=47	n=31	n=7	
**DSA characteristics**				
MFI of the immunodominant DSA, median (IQR)	3,470 (1,753–9,602)	2,239 (1,360–4,837)	1,508 (1,087–1,946)	0.071
**Biopsy results ^a,b^**				
Banff ABMR, n (%)	33 (70)	14 (45)	2 (29)	0.024
g+ptc score^c^, median (IQR)	2 (0–4)	0 (0–2)	0 (0–1)	0.006
MMDx results				
ABMR archetype, n (%)	27 (60)	13 (43)	1 (14)	0.052
ABMR score^c^, median (IQR)	0.43 (0.14–0.72)	0.23 (0.08–0.60)	0.11 (0.10–0.22)	0.043
NK cell burden-associated PBT (NKB), median (IQR)	1.01 (0.50–1.35)	0.61 (0.43–1.22)	0.43 (0.32–0.69)	0.074
IFN-γ-associated PBT (GRIT1), median (IQR)	0.79 (0.46–0.98)	0.57 (0.29–0.94)	0.28 (-0.10–0.44)	0.012

ABMR, antibody-mediated rejection; DSA, donor-specific antibody; g, glomerulitis; IQR, interquartile range; MFI, mean fluorescence intensity; PBT, pathogenesis-based transcripts; ptc, peritubular capillaritis.

a Morphologic lesions were scored according to the Banff 2017 classification of renal pathology.

**^b^** Gene expression analysis was performed in 83 of the 86 study patients.

c For 2 and 4 recipients, biopsy material was not sufficient for ptc, and g scoring, respectively.

### Cohort 2 – Patients Randomly Selected From the Multicenter CTS Database

As detailed in **Table 2**, 725 (39%) of the 1,860 CTS recipients were female, 1,620 (87%) were first transplant recipients, and 412 (24%) had detectable cytotoxic panel reactivity before transplantation. Ninety-five percent of the subjects received initial immunosuppression with a calcineurin inhibitor (cyclosporine A, 81%; tacrolimus, 14%), 47% received azathioprine, and 33% mycophenolic acid. Thirty-one percent of the recipients were given antibody induction (IL-2 receptor antibody, 8%; depleting anti-lymphocyte antibody, 23%). Genotype frequencies of all 3,720 typed DNAs did not deviate from Hardy–Weinberg equilibrium (*P*=0.27). As shown in **Figure 2**, distributions of *KLRC2* variants differed significantly between recipient and donor populations (*P*=0.004). Among recipients, 1,247 (67%) encoded for *KLRC2* ^wt/wt^, 530 (29%) for *KLRC2* ^wt/del^, and 83 (5%) for *KLRC2* ^del/del^. Among donors, 1167 (63%) had the *KLRC2* ^wt/wt^, 622 (33%) the *KLRC2* ^wt/del^ and 71 (4%) the *KLRC2* ^del/del^ genotype (**Figure 2**). Baseline characteristics of recipients were not different between genotypic groups, with the exception of azathioprine-based immunosuppression and cold ischemia time (**Table 2**).

#### KLRC2 Genotype and Transplant Outcomes

**Figure 5:**
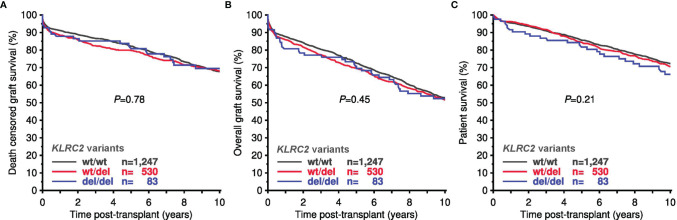
10-year death-censored graft survival **(A)**, overall graft survival **(B)**, and patient survival **(C)** in the Collaborative Transplant Study (CTS) cohort in relation to *KLRC2* genotype (total 1,860 recipients).

As shown in **Figure 5**, Kaplan Meier analyses revealed no significant differences in death-censored allograft survival, overall graft survival, or patient survival in relation to recipient or donor *KLRC2* variants. Also, in multivariable Cox regression analysis no effect of *KLRC2* genotype was observed (**Table 5**). Furthermore, the *KLRC2* polymorphism was not associated with impaired allograft function at one year or an increased need for anti-rejection treatment (**Supplementary Figure 2**). We then focused on recipients with PRA >0% at the time of transplantation, recipients with 4–6 HLA mismatches, and recipients treated for rejection during the first year, hypothesizing a particular role of NK cell-triggered graft injury in these patients. As shown in **Supplementary Figure 3** and **Table 5**, in these subgroups at risk, the *KLRC2* genotype was also not associated with death-censored graft survival.

**Table 5:** Multivariable Cox regression analysis for influence of number of *KLRC2*-wild type alleles on 10-year graft and patient survival in the CTS cohort.

Population	Patients	Death-censored graft survival		Overall graft survival		Patient survival	
		HR (95% CI)	*P*	HR (95% CI)	*P*	HR (95% CI)	*P*
All patients	1,860	0.94 (0.81–1.10)	0.44	0.95 (0.84–1.07)	0.38	0.91 (0.78–1.06)	0.24
PRA>0%	412	0.97 (0.68–1.40)	0.89	0.92 (0.69–1.23)	0.57	0.88 (0.61–1.27)	0.50
4–6 mismatches^a^	680	0.85 (0.66–1.09)	0.20	0.86 (0.71–1.04)	0.11	0.83 (0.65–1.06)	0.14
1-year-rej	227	0.95 (0.59–1.53)	0.83	0.91 (0.62–1.35)	0.65	0.78 (0.46–1.31)	0.35

CTS, Collaborative Transplant Study; PRA, panel-reactive antibodies; rej, rejection treatment.

a HLA-A+B+DR mismatch.

Hazard ratios (HR) and 95% confidence interval (CI) per wild type allele are shown.

#### Combined Analysis of KLRC2 and FCGR3A Genotypes

**Figure 6:**
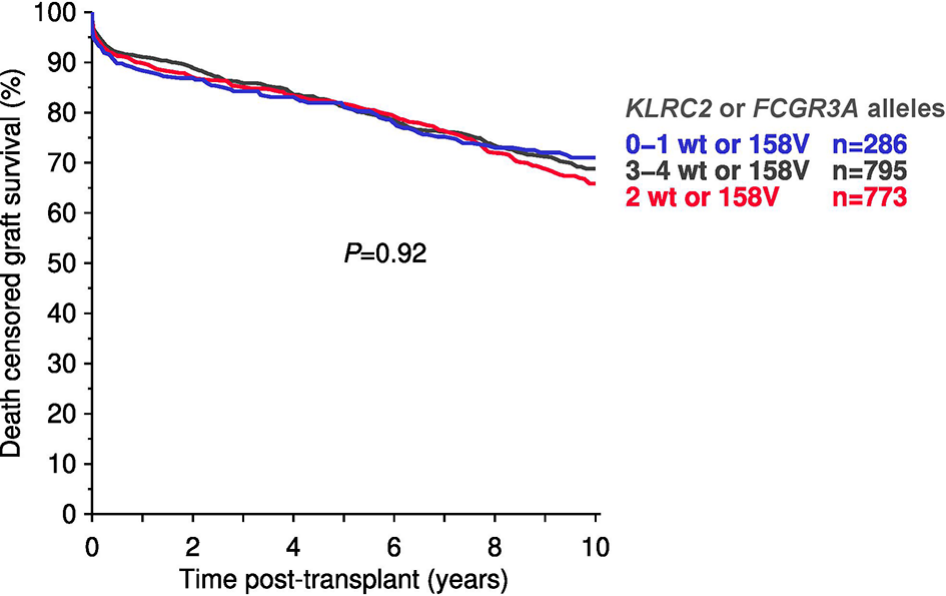
Total number of ‘risk alleles’ (*KLRC2* wild type (wt) and/or the *FCGR3A*-V158 polymorphism) in the Collaborative Transplant Study (CTS) cohort in relation to 10-year death-censored graft survival (total 1,854 recipients.

Of the 1,860 CTS recipients 254 (14%) had V/V158, 845 (46%) V/F158, and 755 (41%) F/F158 genotypes (6 without genotyping results). We have previously shown no death-censored graft survival differences between genotypic groups (11). As shown in **Figure 6**, combined analysis of *KLRC2* and *FCGR3A* variants revealed no significant survival differences in relation to the number of ‘risk genotypes’ (*KLRC2* wild type or *FCGR3A*-V158). No significant effect was observed in multivariable Cox regression analysis for the influence of the sum of *KLRC2* and *FCGR3A* risk alleles on 10-year death-censored graft survival: HR=0.98 (95% CI 0.89–1.08, *P*=0.67) (data not shown).

## Discussion

A key result of this study was that, in a selected cohort of DSA+ renal allograft recipients, *KLRC2* variants known to determine the number and functionality of NKG2C+ NK cells (18–20), were associated with ABMR (but not TCMR) development and morphologic/molecular rejection activity; the latter included NK cell burden-, IFN-y- and macrophage-related transcripts, while morphologic and molecular features of T cell-mediated rejection were not different between genotypic groups. Chronic glomerular injury (cg) and 5-year death-censored graft survival, however, were not significantly different between *KLRC2* ^wt/wt^ *and KLRC2* ^wt/del^ variants. In addition, in a large prospective transplant cohort randomly selected from the CTS database, our findings obtained in the smaller DSA+ cohort did not translate into meaningful differences in long-term allograft survival. There were also no associations of *KLRC2* genotype with other important clinical endpoints, such as graft function or treatment for rejection, and graft survival remained unaffected even in subgroups deemed to be at particular risk of ABMR.

Our results obtained in the DSA+ cohort are in line with a previously proposed role of NK cells in the pathogenesis of ABMR (4–8). Microarray studies have suggested a critical involvement of FcγRIIIA-triggered NK cell cytotoxicity and IFN-γ release (6, 7). These data were supported by two recent studies (one performed with the BORTEJECT cohort which is also analyzed in this study), suggesting a contribution of FcγRIIIA functionality - determined by a *FCGR3A* V/F158 polymorphism - to DSA-triggered MVI (9, 10). Alternative pathways of NK cell activation in organ transplantation, however, may act independently of DSA, including recognition of missing self HLA *via* a set of inhibitory KIR receptors (12, 13). The molecular mechanisms behind a potential pathogenetic contribution of NKG2C (and the studied *KLRC2* variation) remain a matter of speculation. Major triggers of NKG2C activation may be viral (for example CMV-derived) peptides bound to HLA-E. HLA-E, however, may instead bind peptides derived form leader sequences of classical (HLA-C) or non-classical HLA molecules (HLA-G), whereby individual sequences may exert varying activating potential (31). Transcriptomics studies have previously shown that transplant rejection is dominated by transcripts that are IFN-gamma-inducible, and these include also HLA-E (32). Regarding our observation of increased IFN-γ-associated PBT scores and higher HLA-E (but not HLA-G) expression in *KLRC2* ^wt/wt^ subjects, one may speculate that upregulated expression of HLA-E (and perhaps binding of HLA-G-derived peptides) has promoted activation of NKG2C+ cells, the peripheral blood counts of which are known to critically depend on the number of *KLRC2* wild type genes. In addition, it was previously shown that NKG2C^+^ NK cells may exert strong effector functions *via* FcγR engagement (33), and this could at least partly explain the observed interaction between *KLRC2 and FCGR3A*-V/F158 genotypes.

A remarkable observation for which we have no meaningful explanation is the significantly higher DSA levels (as reflected by MFI) among *KLRC2* ^wt/wt^ recipients. A causative relationship between NK cell function/counts, upregulation of HLA molecules, and the development of donor-specific B cell alloimmunity can be speculated, but will need validation in independent patient cohorts. Moreover, distributions of *KLRC2* genotypes among transplant recipients differed significantly from those observed in matched DSA– control patients (DSA+ recipients; cohort 1) or organ transplant donors (CTS recipients; cohort 2), with higher proportions of *KLRC2* ^wt/wt^ patients found among study patients. We have no clear explanation for the shift in genotype distribution, but one may speculate regarding associations of the *KLRC2* ^wt/wt^ variant with the risk of kidney failure or the susceptibility to HLA sensitization. To our best knowledge, there are no previous reports suggesting associations between distinct genetic determinants of NK cell-driven alloresponses, such as KIR genotype and missing self (13), and DSA formation. We are aware of the preliminary nature of our results, which need to be interpreted with caution.

In our study cohort, the majority of the recipients had a high or intermediate-risk CMV constellations at the time of transplantation, with no significant difference between *KLRC2* genotypic groups. We can speculate regarding how previous exposure to CMV may contribute to NK cell activity. Earlier studies have suggested that CMV infection may play a role in triggering allograft rejection (34, 35), even though the mechanisms contributing to alloimmunity are not well understood. In addition to the potential role played by cross-reactive T cells (36), we can hypothesize regarding the role of enhanced NK cell responsiveness. In this respect, a recent experimental study may be of interest. In this study not only NK cell antibody-dependent cell mediated cytotoxicity (ADCC), but also non-ADCC alloreactivity against HLA-positive target cells, was found to be increased in CMV seropositive when compared to CMV seronegative healthy individuals. Seropositivity was associated with an increase in the number of NKG2C+ cells, and the authors speculated that a CMV-triggered change in activation threshold levels could facilitate NK cell activation *via* DSA or missing-self (37). HLA-E upregulation in rejecting allografts and presentation of distinct variants of virally encoded UL40 peptides may thereby contribute to NK cell-triggered graft injury, as has been shown for lung transplant recipients who developed chronic lung allograft dysfunction (38, 39).

In the large CTS cohort, we found no association between KLRC2 genotype and long-term transplant outcomes; this was the case even in patients at particularly high risk of ABMR (recipients with detectable anti-HLA antibodies at the time of transplantation, a high HLA mismatch, or rejection treatment within the first year post-transplant). These data suggest that *KLRC2* genotyping may have no relevance for risk-stratification in unselected transplant cohorts. This may be due to the overwhelming effect of a variety of different immunologic and non-immunologic primary or secondary causes of graft loss (40). Discussing the apparent discrepancy between associations with morphologic and molecular biopsy results among DSA-positive recipients and the observed lack of long-term survival differences in the two studied cohorts, we want to mention a recent study of 924 renal allograft recipients suggesting an important role of KIR/HLA genotype and missing self as a trigger of NK cell-dependent MVI (13). Despite being strongly associated with MVI and structural alterations to the allograft microvasculature, missing self *per se* turned out to be not associated with graft survival rates. The authors discussed missing self/NK cell-driven graft injury as a more transient or weaker phenomenon than DSA-mediated organ damage. Their results were thereby in line with a study by Tran et al. (41) who found no impact of KIR-ligand mismatching on 10-year graft survival in a CTS-derived multicenter cohort of 608 recipient/donor pairs.

While a large sample size and thus sufficient power to detect subtle outcome differences may be a major strength, we are aware of several inherent limitations. Most importantly, these include the lack of detailed HLA antibody data, including donor specificity and granular allograft biopsy results to assess the extent of MVI or distinct rejection phenotypes, in particular, TCMR and ABMR. Prior to 2000, ABMR criteria, as well as solid phase single bead HLA antibody analysis, were not established. Therefore, this information was not available for our CTS cohort. We are aware that in our subanalysis of patients with detectable panel reactivity based on CDC testing, many had not been exposed to DSA either before or after transplantation, and were thus not at risk of ADCC-mediated graft injury. Similarly, only a minor proportion of patients treated for rejection may have had DSA-triggered ABMR, with a majority of them experiencing TCMR without any detectable DSA.

In conclusion, the results of our study are suggestive of an influence of NKG2C expression in the context of allograft rejection, as supported by increased levels of MVI and ABMR-related gene expression patterns among DSA+ patients harboring the *KLRC2* ^wt/wt^ genotype. However, the results obtained in our DSA+ cohort did not translate into graft survival differences. Even in a large randomly selected transplant cohort powered to detect small differences in transplant outcomes, we did not find any survival effect. This was also the case in sub-analyses of patients at particular risk of ABMR. We are aware that our results need to be interpreted with caution, but our results may provide a valuable basis for large, adequately powered studies designed to include granular endpoints, such as the results of sequential surveillance biopsies, in order to clarify the actual role of genetically determined NKG2C expression and functionality as a risk factor for NK cell-driven alloimmune injury in kidney transplant recipients. In this context, a comprehensive, combined analysis including different genetic determinants of NK cell activity, in addition to functional NKG2C and FcγRIIIA gene variants, such as KIR-ligand mismatches or polymorphisms/haplotypes determining the functionality of other important receptors (42), would contribute significantly to a better understanding of NK cell biology in transplant medicine.

## Data Availability Statement

The original contributions presented in the study are included in the article/**Supplementary Material**. Further inquiries can be directed to the corresponding authors.

## Ethics Statement

The studies involving human participants were reviewed and approved by Ethics Committees of the University of Heidelberg and the Medical University of Vienna. The patients/participants provided their written informed consent to participate in this study.

## Author Contributions

HV, BD, CS, EP-S, and GB: participated in the research design, performance of the research, data analysis, interpretation of results, and writing of the manuscript. TT, PH, FE, CH, KM, and NK: participated in data analysis and writing of the manuscript. MW and SH: participated in performance of the research. SE: participated in writing of the manuscript. All authors contributed to the article and approved the submitted version.

## Funding

The trial was funded by a grant from the Austrian Science Fund (KLI190 to GB).

## Conflict of Interest

The authors declare that the research was conducted in the absence of any commercial or financial relationships that could be construed as a potential conflict of interest.

## Publisher’s Note

All claims expressed in this article are solely those of the authors and do not necessarily represent those of their affiliated organizations, or those of the publisher, the editors and the reviewers. Any product that may be evaluated in this article, or claim that may be made by its manufacturer, is not guaranteed or endorsed by the publisher.

## Abbreviations

ABMR, antibody-mediated rejection; ADCC, antibody-dependent cellular cytotoxicity; CDC, complement-dependent cytotoxicity; cg, glomerular basement membrane double contours; CI, confidence interval; CMV, cytomegalovirus; CTS, Collaborative Transplant Study; DSA, donor-specific antibodies; eGFR, estimated glomerular filtration rate; FcγR, Fc gamma receptor; g, glomerulitis; eGFR, estimated glomerular filtration rate; HR, hazard ratio; IQR, interquartile range; KIR, killer-cell immunoglobulin-like receptor; MFI, mean fluorescence intensity; MMDx, Molecular Microscope Diagnostic System; MVI, microvascular inflammation; NK cell, natural killer cell; PBT, pathogenesis-based transcripts; ptc, peritubular capillaritis; SD, standard deviation; SNP, single nucleotide polymorphism.
