# Supplementary material for: Deletion of the Natural Killer Cell Receptor NKG2C Encoding KLR2C Gene and Kidney Transplant Outcome
Source: Front Immunol. 2022 Mar 24;13:829228. doi: 10.3389/fimmu.2022.829228 (PMC8987017; doi:10.3389/fimmu.2022.829228)
Supplement: Supplementary file 1 [file Image_1.pdf]

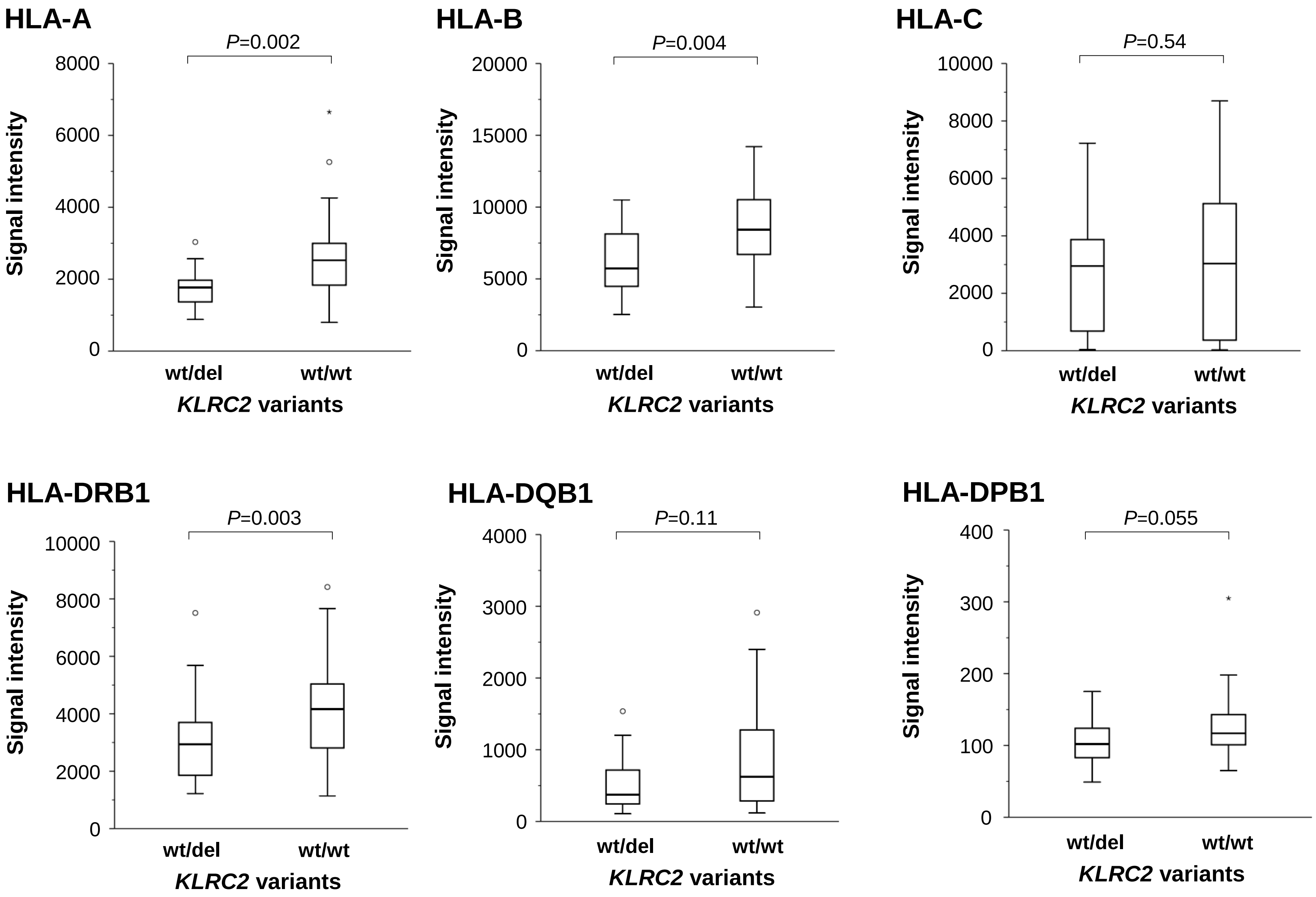

Supplemental Figure 1. *KLRC2* polymorphism in the DSA+ BORTEJECT cohort in relation to intragraft HLA class I (HLA-A, HLA-B and HLA-C) and HLA class II (HLA-DRB1, HLA-DQB1, HLA-DPB1) transcript expression (microarray signal intensity).
