# Supplementary material for: Deletion of the Natural Killer Cell Receptor NKG2C Encoding KLR2C Gene and Kidney Transplant Outcome
Source: Front Immunol. 2022 Mar 24;13:829228. doi: 10.3389/fimmu.2022.829228 (PMC8987017; doi:10.3389/fimmu.2022.829228)
Supplement: Supplementary file 2 [file Image_2.pdf]

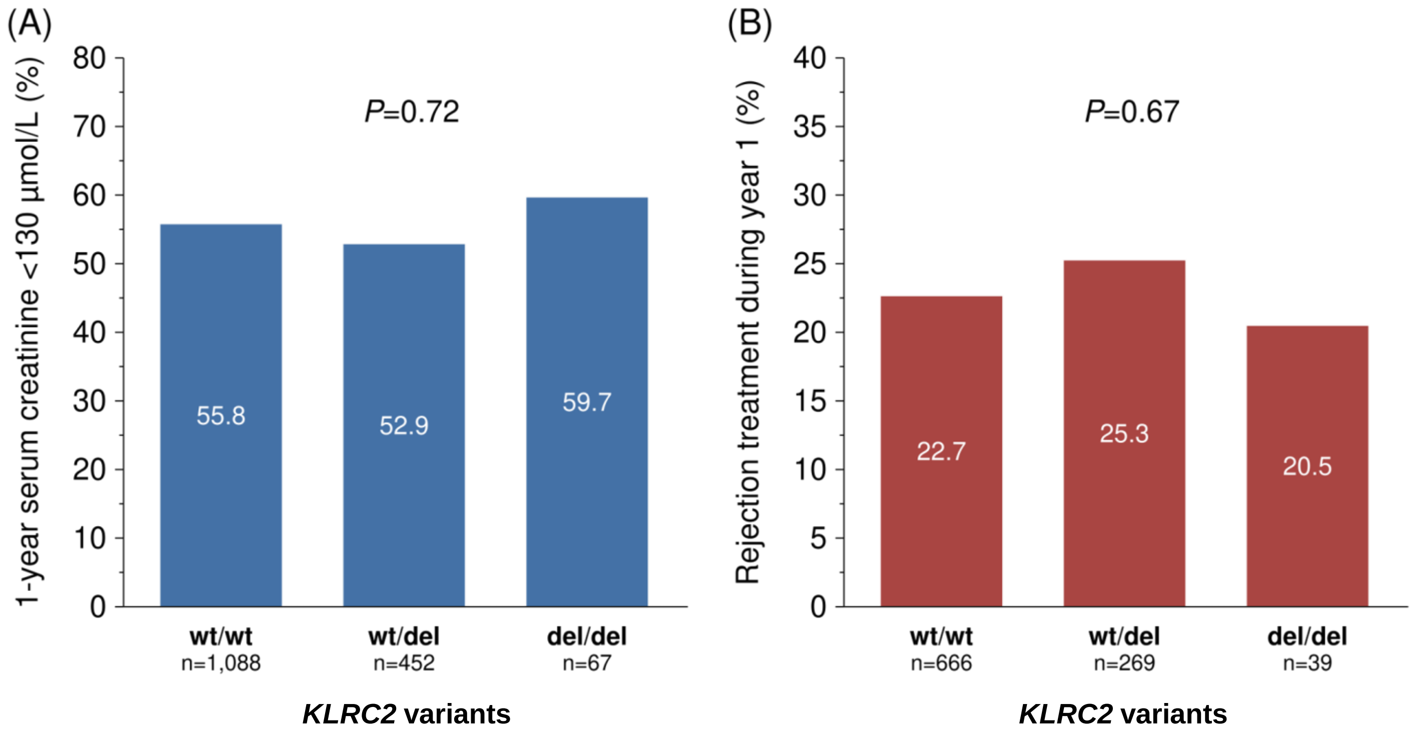

Supplemental Figure 2. *KLRC2* polymorphism in the CTS cohort in relation to 1-year serum creatinine (A; total 1,607 recipients) and treated rejection episodes within the first year after transplantation (B; total 974 recipients).
