# Supplementary material for: Deletion of the Natural Killer Cell Receptor NKG2C Encoding KLR2C Gene and Kidney Transplant Outcome
Source: Front Immunol. 2022 Mar 24;13:829228. doi: 10.3389/fimmu.2022.829228 (PMC8987017; doi:10.3389/fimmu.2022.829228)
Supplement: Supplementary file 3 [file Image_3.pdf]

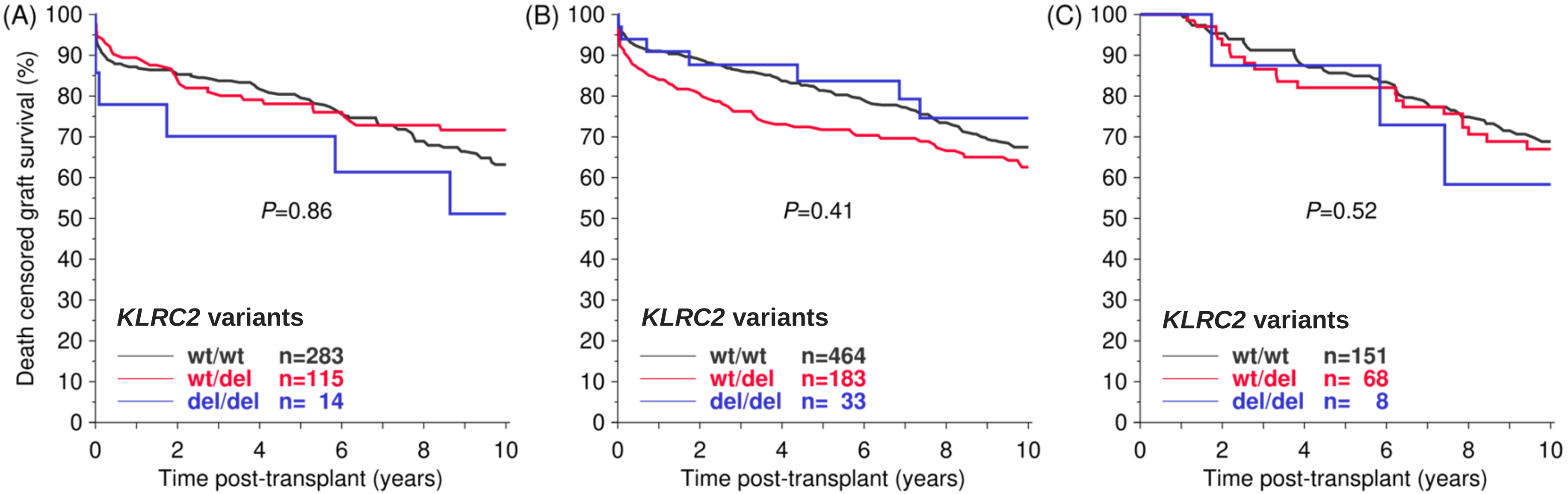

Supplemental Figure 3. Death-censored graft survival in the CTS cohort among (A) 412 pre-sensitized recipients (panel reactivity > 0%), (B) 680 recipients with 4–6 HLA-A+B+DR mismatches, and (C) 227 recipients with treated rejection during the first-post-transplant year, in relation to *KLRC2* genotype.
