# Supplementary material for: Deletion of the Natural Killer Cell Receptor NKG2C Encoding KLR2C Gene and Kidney Transplant Outcome
Source: Front Immunol. 2022 Mar 24;13:829228. doi: 10.3389/fimmu.2022.829228 (PMC8987017; doi:10.3389/fimmu.2022.829228)
Supplement: Supplementary file 4 [file Table_1.pdf]

SUPPLEMENTAL TABLE 1. Baseline characteristics – DSA+ versus DSA– transplant cohorts (BORTEJECT)

Parameters	DSA+ (study patients) n=86	DSA– (matched control)[a] n=106	*P* - value
**Characteristics obtained at the time of transplantation**			
Female sex, n (%)	39 (45)	45 (43)	0.77
Recipient age (years), median (IQR)	47 (36-54)	48 (38-56)	0,63
First renal allograft, n (%)	61 (71)	69 (65)	0.44
Living donor transplantation, n (%)	14 (16)	18 (17)	>0.99
Donor age (years)[b], median (IQR)	46 (35-58)	48 (35-56)	0.99
Cold ischemia time (hours)[b], median (IQR)	12 (9-17)	13 (9-18)	0.25
HLA mismatch (A, B, DR)[b], median (IQR)	3 (2-4)	3 (2-4)	0.98
CDC-PRA ≥0%[b], n (%)	30 (37)	39 (39)	0.88
Preformed DSA[c], n (%)	25 (60)	19 (40)	0.09
ABO-incompatible living donor transplant[d], n (%)	1 (1)	0 (0)	0.45
Recipient desensitization, n (%)	26 (30)	19 (18)	0.059
CDC crossmatch conversion, n (%)	8 (9)	4 (4)	0.14
**Characteristics obtained at the time of ABMR screening**			
Time to screening (years), median yrs (IQR)	4.9 (1.9–12.8)	5.9 (2.9–10.6)	0.67
Serum creatinine (mg/dL), median (IQR)	1.6 (1.2–2.1)	1.4 (1.2–1.9)	0.11
eGFR (mL/min/1.73m^2), median (IQR)	54 (32–79)	63 (39–88)	0.074
Urinary protein/creatinine ratio (mg/g), median (IQR)	192 (79–445)	156 (83–453)	0.84
Maintenance immunosuppression			
Triple immunosuppression	65 (76)	81 (76)	>0.99
Dual immunosuppression	21 (24)	25 (24)	>0.99
Tacrolimus	52 (60)	76 (72)	0.12

Cyclosporine A	29 (34)	25 (24)	0.15
mTOR Inhibitor	4 (5)	3 (3)	0.70
Belatacept	1 (1)	1 (1)	>0.99
Mycophenolic acid	71 (83)	85 (80)	0.71
Azathioprine	5 (6)	8 (8)	0.78
Steroids	75 (88)	92 (87)	>0.99

CDC, complement-dependent cytotoxicity; DSA, donor-specific antibody; eGFR, estimated glomerular filtration rate; HLA, human leukocyte antigen; IQR, interquartile range; mTOR, mammalian target of rapamycin; PRA, panel-reactive antibody.

[a]The cohort of DSA– recipients was propensity score matched to DSA+ study patients (variables: female sex, recipient age, urinary protein/creatinine ratio, prior transplantation, HLA mismatch and cytotoxic panel reactivity).

[b]Donor age, cold ischemia time, HLA mismatch and CDC panel reactivity were not recorded for 3, 5, 1 and 5 DSA+ and 1, 8, 1 and 5 DSA– recipients, respectively.

[c]Pre-transplant single antigen testing was available for 42 DSA+ and 47 DSA– patients (solid-phase HLA antibody screening on the wait list according to our local standard implemented in July 2009). Pre-sensitized patients (until 2009: ≥40% CDC-PRA; since 2009: preformed DSA) were subjected to a protocol of peri-transplant immunoadsorption as earlier detailed.

[d]This patient underwent desensitization for ABO (AB donor to O recipient) plus HLA antibody (DSA+) barriers.
